# RDR2 Partially Antagonizes the Production of RDR6-Dependent siRNA in Sense Transgene-Mediated PTGS

**DOI:** 10.1371/journal.pone.0029785

**Published:** 2012-01-05

**Authors:** Vincent Jauvion, Maud Rivard, Nathalie Bouteiller, Taline Elmayan, Hervé Vaucheret

**Affiliations:** Institut Jean-Pierre Bourgin, INRA, Versailles, France; University of Leeds, United Kingdom

## Abstract

**Background:**

RNA-DEPENDENT RNA POLYMERASE6 (RDR6) and SUPPRESSOR of GENE SILENCING 3 (SGS3) are required for DNA methylation and post-transcriptional gene silencing (PTGS) mediated by 21-nt siRNAs produced by sense transgenes (S-PTGS). In contrast, RDR2, but not RDR6, is required for DNA methylation and TGS mediated by 24-nt siRNAs, and for cell-to-cell spreading of IR-PTGS mediated by 21-nt siRNAs produced by inverted repeat transgenes under the control of a phloem-specific promoter.

**Principal Findings:**

In this study, we examined the role of RDR2 and RDR6 in S-PTGS. Unlike RDR6, RDR2 is not required for DNA methylation of transgenes subjected to S-PTGS. RDR6 is essential for the production of siRNAs by transgenes subjected to S-PTGS, but RDR2 also contributes to the production of transgene siRNAs when RDR6 is present because *rdr2* mutations reduce transgene siRNA accumulation. However, the siRNAs produced via RDR2 likely are counteractive in wildtype plants because impairement of RDR2 increases S-PTGS efficiency at a transgenic locus that triggers limited silencing, and accelerates S-PTGS at a transgenic locus that triggers efficient silencing.

**Conclusions/Significance:**

These results suggest that RDR2 and RDR6 compete for RNA substrates produced by transgenes subjected to S-PTGS. RDR2 partially antagonizes RDR6 because RDR2 action likely results in the production of counteractive siRNA. As a result, S-PTGS efficiency is increased in *rdr2* mutants.

## Introduction

Most Eukaryotic genomes produce small RNAs, 20 to 30 nucleotides (nt) in length, which regulate endogenous genes at either the transcriptional or posttranscriptional level [1], [2], [3]. Endogenous small RNA species fall into three major classes: microRNAs (miRNAs) and short-interfering RNAs (siRNAs), which are both produced by Dicer enzymes, and piwi-related RNAs (piRNAs), which are Dicer-independent. All classes of small RNAs associate with proteins of the Argonaute/Piwi family [4]. Exogenous siRNAs can also be produced in response to invasive DNA or RNA (transgenes, viruses, bacteria, etc). This *de novo* production of siRNAs relies on the existing cellular small RNA machineries [5]. In contrast to endogenous small RNAs that usually are specifically processed from their precursor RNAs by one or the other cellular machinery, exogenous precursor RNAs can be processed into various forms of siRNAs by the different cellular machineries. These different ways to process exogenous RNAs have different silencing outcomes. For instance, in plants, 21-nt and 22-nt siRNAs produced by DCL4 and DCL2, respectively, trigger posttranscriptional gene silencing (PTGS) when they are homologous to transcribed regions, either by guiding mRNA cleavage or translational repression [6]. In contrast, 24-nt siRNAs produced by DCL3 trigger transcriptional gene silencing (TGS) when they are homologous to promoter regions, either by guiding DNA methylation or histone modification [7].

What makes an exogenously derived double-stranded RNA (dsRNA) a particularly attractive substrate for one DCL or another remains unclear. When the exogenous dsRNA is directly transfected into the cell or produced in the cell in the form of a dsRNA, only its sequence and structure could account for DCL specificity. However, when the dsRNA derives from an exogenous single-stranded RNA (ssRNA) transformed into dsRNA by an endogenous RNA-dependent RNA polymerase (RDR), the DCL specificity could rely on the specific relationship existing between RDRs and DCLs. It is known that in wildtype plants, DCL4 processes endogenous RDR6-derived dsRNA [8], whereas DCL3 processes endogenous RDR2-derived dsRNA [9]. In contrast, DCL2 mostly processes RDR-independent dsRNA produced by endogenous inverted repeats [10]. DCL2, DCL3 and DCL4 can substitute to each other when one is missing [8] and only when DCL2, DCL3 and DCL4 are missing can DCL1 process some siRNAs in addition to miRNAs [11].

Although RDR6 and DCL4 normally function in PTGS while RDR2 and DCL3 function in TGS, several unexpected requirements have been observed during transgene silencing. First, only RDR2, but not RDR6, functions with DCL4 in cell-to-cell spreading of IR-PTGS, i.e. PTGS triggered by inverted repeat transgenes, when the primary transcript is expressed in a localized tissue-specific manner [12], [13]. Secondly, RDR6 is required for DNA methylation of transcribed regions during S-PTGS i.e. PTGS triggered by sense transgenes [14]. In this report, we examined the roles of RDR2 and RDR6 in S-PTGS. We found that RDR2 is not required for DNA methylation of transcribed regions during S-PTGS, suggesting that this type of methylation is mostly triggered by RDR6-dependent 21-nt siRNAs. We also found that S-PTGS is more efficient in *rdr2* mutants than in wildtype plants, suggesting that RDR2 partially antagonizes RDR6 during the triggering of S-PTGS.

## Results

### CLSY1, NRPD1 and RDR2 are not required for S-PTGS

Genetic screens were designed to identify cellular components involved in the initiation or spreading of IR-PTGS. Arabidopsis transgenic lines expressing IR-transgenes that generate dsRNA under the control of the *SUCROSE2* (*SUC2*) promoter, which drives expression specifically in the companion cells of the phloem, were generated to specifically search for mutants impaired in the spreading of PTGS. Tested dsRNA inducers included those that targeted *SULPHUR* (*SUL*) and *PHYTOENE DESATURASE* (*PDS*), which, when silenced, lead to bleaching of the leaf tissue. These IR-PTGS lines exhibited silencing of the *SUL* and *PDS* targets in a layer of 10–15 cells around the vasculature, due to the spreading of a mobile PTGS signal. Silencing in the *pSUC2:SUL* and *pSUC2:PDS* lines was not impaired by mutations in *RDR6* and *SGS3* [10], [13], [15]. Mutagenesis of the *pSUC2:SUL* and *pSUC2:PDS* lines retrieved mutants impaired in the chromatin-remodeling protein CLSY1, the largest (NRPD1) and second largest (NRPD2) subunits of PolIV and the RNA-dependent RNA polymerase RDR2 [10], [12], [13].

**Figure 1:**
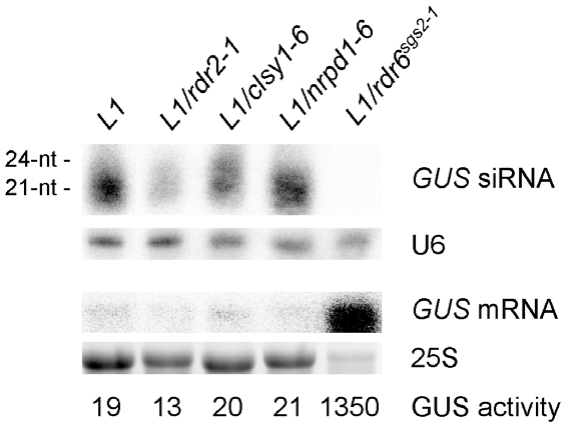
RNA gel blot analysis of *L1*, *L1/clsy1*, *L1/nrpd1*, *L1/rdr2* and *L1/rdr6* plants. Null alleles of *clsy1*, *nrpd1*, *rdr2* and *rdr6* were used in this analysis. LMW and HMW RNA gel blot of 11-day-old seedlings of the indicated mutant plants was probed with an RNA GUS probe. U6 snRNA and 25S rRNA hybridizations served as a loading control. GUS activity quantification (in fluorescence units per min and per ug of total protein).

Neither *clsy1, nrpd1* nor *rdr2* mutants were recovered from the *L1* forward genetic screen. Therefore, we examined the integrity of the S-PTGS pathway when CLSY1, NRPD1 and RDR2 are impaired by crossing *L1* to null alleles of *clsy1*, *nrpd1* and *rdr2*. F2 plants that are homozygous for both L1 and the *clsy1*, *nrpd1* or *rdr2* mutations were identified, and bulks of F3 plants were analyzed 11 days after germination (DAG). *L1/rdr6* was used as a control for the absence of *GUS* S-PTGS. *GUS* mRNA and GUS activity in *L1/clsy1*, *L1/nrpd1* and *L1/rdr2* were low and comparable to control *L1* plants (Figure 1), indicating that neither of these mutations delayed the onset of S-PTGS or compromised its establishment. Consistently, *GUS* siRNAs accumulated in *L1/clsy1*, *L1/nrpd1* and *L1/rdr2*, although at levels lower than in *L1* (Figure 1). These results indicate that CLSY1, NRPD1 and RDR2, which are required for the production of siRNAs associated to the spreading of IR-PTGS, are dispensable for S-PTGS.

### CLSY1, NRPD1 and RDR2 are not required for DNA methylation in S-PTGS

**Figure 2:**
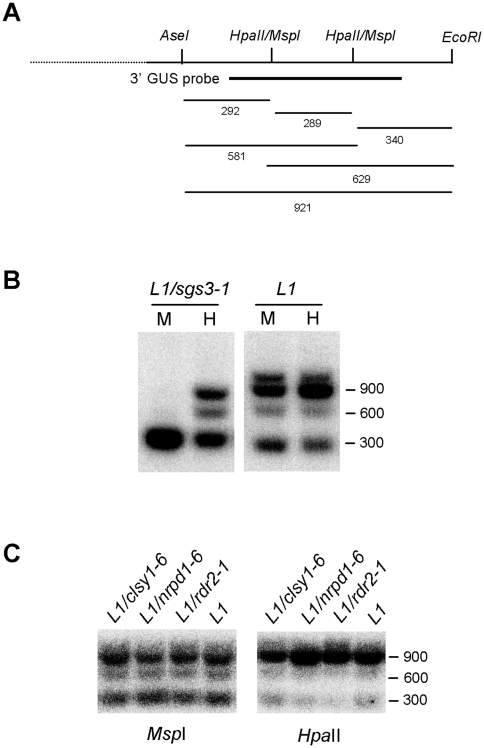
DNA gel blot analysis of *L1*, *L1/clsy1*, *L1/nrpd1*, *L1/rdr2* and *L1/sgs3* plants. A) Partial map of the GUS coding sequence and expected digestion fragments. Methylation insensitive *AseI*, *EcoRI* and methylation sensitive *HpaII/MspI* sites are indicated, as well as the expected digestion fragments hybridizing with the 3′GUS probe (indicated in bold). B) DNA of 11-day-old *L1* and *L1/sgs3* seedlings was digested with *AseI*, *EcoRI* and *MspI* (indicated as M) or *AseI*, *EcoRI* and *HpaII* (indicated as H) and probed with a DNA 3′ GUS probe. C) DNA of 11-day-old *L1*, *L1/clsy1*, *L1/nrpd1* and *L1/rdr2* seedlings was digested with *AseI*, *EcoRI* and *MspI* or *HpaII* and probed with a DNA 3′ GUS probe. The *clsy1*, *nrpd1* and *rdr2* material used in this analysis was similar to that in Figure 1.

CLSY1, NRPD1 and RDR2 are required for RNA-directed DNA methylation (RdDM) guided by endogenous 24-nt siRNAs [9], [16], [17]. As such, they are required for RdDM of the *FWA* transgene and endogenous gene [16], [18], and for RdDM of the endogenous *PDS* locus in *pSUC2:PDS* lines [19]. DNA methylation also is a hallmark of S-PTGS, as examplified by *GUS* DNA methylation in the *L1* line [20], which requires RDR6 and SGS3 [14]. Indeed, methylation at CNG sites (monitored by *MspI* digest) in the 3′ end of *GUS* is abolished when S-PTGS is compromised (Figure 2B). However, it is not known if *GUS* DNA methylation occurs at CNG sites because RDR6 and SGS3 produce siRNA that directly guide DNA methylation at CNG sites or because RDR6 and SGS3 produce siRNA that are used by CLSY1, NRPD1 or RDR2 to produce secondary molecules that guide DNA methylation. Moreover, methylation at CG sites (monitored by *HpaII* digest) is strongly reduced but not abolished when S-PTGS is compromised (Figure 2B), suggesting that *GUS* DNA methylation at CG sites is partly independent of RDR6 and SGS3. Whether, RDR6-SGS3-independent DNA methylation at CG sites requires the CLSY1, NRPD1 and RDR2 components of RdDM is unknown.

To address the role of CLSY1, NRPD1 and RDR2 in *GUS* DNA methylation in the *L1* line, DNA methylation of the *GUS* transgene was examined at 11 DAG in *L1/clsy1*, *L1/nrpd1* and *L1/rdr2* mutants. At CNG sites, *GUS* DNA methylation in *L1/clsy1*, *L1/nrpd1* and *L1/rdr2* mutants was comparable to control *L1* plants (Figure 2C), indicating that CLSY1, NRPD1 and RDR2 are dispensable for RDR6-SGS3-dependent DNA methylation in S-PTGS. At CG sites, we expected *GUS* DNA methylation in *L1/clsy1*, *L1/nrpd1* and *L1/rdr2* mutants to be reduced compared to control *L1* plants if CLSY1, NRPD1 and RDR2 contributed to RDR6-SGS3-independent DNA methylation. However, *GUS* DNA methylation in *L1/clsy1*, *L1/nrpd1* and *L1/rdr2* mutants was not reduced compared to control *L1* plants (Figure 2C), indicating that RDR6-SGS3-independent DNA methylation does not require CLSY1, NRPD1 and RDR2 either. The exact pathway contributing to RDR6-SGS3-independent *GUS* DNA methylation at CG sites is still not fully understood, but it likely requires MET1 because *met1* mutants recovered from the *L1* screen exhibit lower levels of *GUS* DNA methylation at CG sites than *rdr6* and *sgs3* mutants [21], [22].

### RDR2 counteracts the production of RDR6-dependent siRNA in S-PTGS

**Figure 3:**
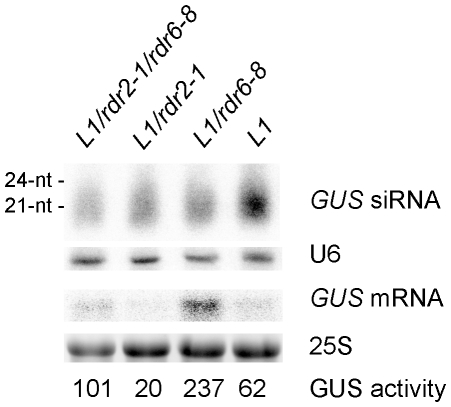
RNA gel blot analysis of *L1*, *L1/rdr2*, *L1/rdr6*, *and L1/rdr2/rdr6* plants. An hypomorphic *rdr6* allele that delays the establishment of S-PTGS was used in this analysis in combination with a nul *rdr2* allele. LMW and HMW RNA gel blot of 8-day-old seedlings of the indicated mutant plants was probed with an RNA GUS probe. U6 snRNA and 25S rRNA hybridizations served as a loading control. GUS activity quantification (in fluorescence units per min and per ug of total protein).

Examination of *GUS* siRNA levels in *L1*, *L1/clsy1*, *L1/nrpd1* and *L1/rdr2* revealed an important reduction of the level of *GUS* siRNAs in *L1/rdr2* compared to *L1* (Figure 1). Moreover, examination of DNA methylation revealed a slightly higher level of DNA methylation at CG sites in *L1/rdr2* compared to *L1* (visible as a slight reduction of the amount of unmethylated fragments on the *HpaII* digest on Figure 2C). Because *L1/clsy1* and *L1/nrpd1* did not show a lower level of *GUS* siRNAs or a higher level of DNA methylation than *L1*, it is unlikely that the impairment of the entire CLSY1/NRPD1/RDR2 pathway was responsible for these phenomena. Rather, we hypothesized that part of the *GUS* siRNAs detected in a wildtype plant was produced by RDR2, independently of CLSY1 and NRPD1. However, it is unlikely that the RDR2-dependent fraction of *GUS* siRNAs plays an active role in *L1* S-PTGS or *GUS* DNA methylation because the impairment of RDR2 activity does not compromise the establishment of S-PTGS and the establishment of *GUS* DNA methylation. Instead, S-PTGS seemed to be established more efficiently in *L1/rdr2* than in *L1* because *GUS* mRNA level and GUS activity at 11 DAG were lower and *GUS* DNA methylation slightly higher in *L1/rdr2* compared with *L1* (Figures 1 and 2). To confirm this hypothesis, we analyzed *GUS* mRNA level and GUS activity at an earlier stage (8 DAG). Indeed, *GUS* mRNA level and GUS activity in *L1* progressively decrease during the first weeks following germination [23], allowing to visualize differences in PTGS efficiency easily when *L1* S-PTGS is not fully established yet. At 8 DAG, *GUS* mRNA level and GUS activity were much lower in *L1/rdr2* compared with *L1* (Figure 3).

We hypothesized that the higher S-PTGS efficiency in *rdr2* mutants could be due to the exclusive synthesis of *GUS* dsRNA by RDR6 in *rdr2* mutants, whereas *GUS* dsRNA are synthesized by both RDR6 and RDR2 in wildtype plants. This hypothesis implies that RDR2-derived *GUS* dsRNA do not play an active role in S-PTGS, which therefore relies only on RDR6-derived *GUS* dsRNA. Supporting this hypothesis, S-PTGS is totally abolished in *rdr6* nul alleles [14], indicating that RDR2 cannot compensate the absence of RDR6. To test further our hypothesis, we generated a double mutant between the *rdr6-8* hypomorphic allele and an *rdr2* nul allele. The *rdr6-8* hypomorphic allele corresponds to a T→A nucleotide change that results in a Y→N amino acid change at protein position 228 [24]. In this mutant, *L1* S-PTGS is not compromised but its establishment is delayed. As a result, *GUS* mRNA level and GUS activity at 8 DAG are higher in *L1/rdr6-8* compared with *L1* (Figure 3). In the *L1/rdr6-8 rdr2* double mutant, *GUS* mRNA and GUS activity were intermediate between *L1/rdr6-8* and *L1*, indicating that RDR2 counteracts the optimum functioning of S-PTGS in the *rdr6-8* hypomorphic allele.

### RDR2 counteracts the triggering of S-PTGS

**Figure 4:**
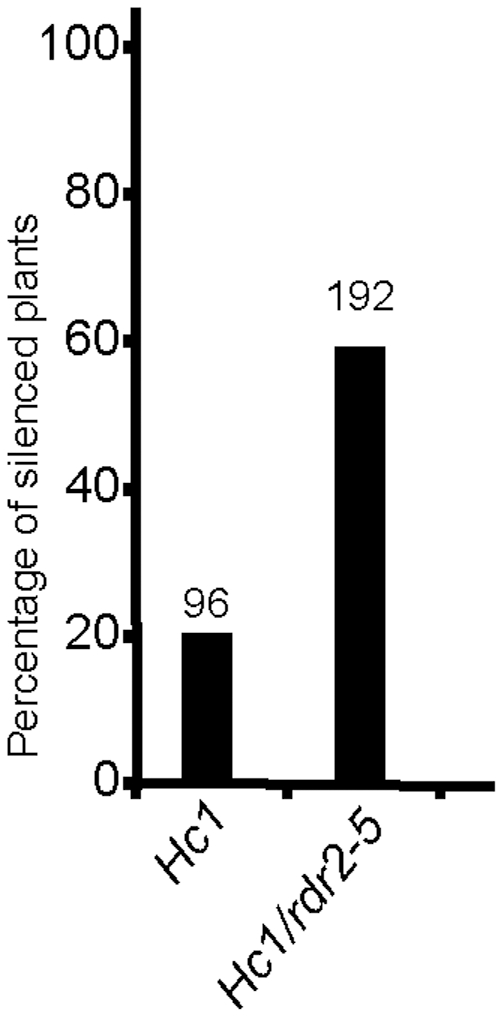
S-PTGS efficiency in *Hc1* and *Hc1/rdr2* plants. A null allele of *rdr2* was used in this analysis. S-PTGS efficiency is reported as the percentage of silenced plants of the indicated lines at 40 days after germination. The number of individual plants analyzed is indicated above each bar.

*rdr2* mutations accelerate the establishment of *L1* S-PTGS in wildtype plants or *rdr6-8* mutants, suggesting that RDR2 counteracts S-PTGS during the triggering or the amplification phase of S-PTGS, or both. To further determine at which step RDR2 competes with RDR6, we introduced the *Hc1* locus into a null *rdr2* mutant. The *Hc1* line carries the same *p35S:GUS* transgene as *L1* but only triggers S-PTGS in 20% of the plants at each generation, whereas *L1* triggers S-PTGS in 100% of the plants [20]. Introduction of the *Hc1* locus into a mutant background therefore allows detecting an increase or a decrease in the triggering of S-PTGS [24], [25]. If RDR2 competed with RDR6 during the triggering phase, we expected the percentage of silenced *Hc1/rdr2* plants to be higher than in *Hc1* plants. However, if RDR2 competed with RDR6 during the amplification phase, we expected S-PTGS to be established faster in *Hc1/rdr2* plants compared with *Hc1* plants, but the percentage of silenced plants to remain the same in *Hc1* and *Hc1/rdr2* plants. GUS activity was determined at the adult stage when silencing is fully established. As previously reported, 19% (18/96) of *Hc1* plants were silenced. In contrast, PTGS affected 56% (107 out of 192) of *Hc1/rdr2* plants (Figure 4), indicating that RDR2 counteracts the triggering of *Hc1* S-PTGS in wildtype plants. This counteracting effect of RDR2 during the triggering phase does not exclude that RDR2 could also counteract the amplification phase of S-PTGS.

## Discussion

Defense responses to invasive DNA or RNA (transgenes, viruses, bacteria, etc) rely on the existing cellular small RNA machineries. What makes an exogenously derived RNA a particularly attractive substrate for one or another machinery remains unclear. In the recent years, it has become clear that endogenous RNAs compete with each other for the accessibility to cellular machineries [8], [11], [26], [27], and that exogenous RNAs also compete with endogenous RNAs [10], [23]. Based on early genetic screens, RDR6 has been associated to 21-nt siRNA-related PTGS mediated by amplicons (A-PTGS) or sense transgenes (S-PTGS) [14], [28], and RDR2 to 24-nt siRNA-related DNA methylation and TGS [9], [18]. However, subsequent genetic screens associated RDR2 to A-PTGS and to IR-PTGS mediated by inverted repeat transgenes [12], [13], [17]. During A-PTGS, RDR2 and RDR6 are partially redundant, at least in some tissues [17], whereas in IR-PTGS, RDR2, but not RDR6, is required for the production of 21-nt and 24-nt siRNAs involved in cell-to-cell spreading of PTGS [12], [13]. Here, we examined the role of RDR2 and RDR6 in S-PTGS and found that both participate in the production of transgene siRNAs. However, the siRNAs produced via RDR2 appear unproductive because S-PTGS is more efficient in *rdr2* mutants than in wildtype plants. These results suggest that RDR2 and RDR6 can compete for S-PTGS RNA substrates, and that RDR2 partially antagonizes the action of RDR6 during S-PTGS. This situation is inverse to that described for Gypsy-like transposons. Indeed, Gypsy-like dsRNA over-accumulate in *rdr6* mutants, suggesting that RDR6 antagonizes the action of other RDR on these targets [27]. The cellular and/or molecular bases of the specificity of RDR proteins towards their RNA substrates remain to be determined.

## Materials and Methods

### Plant material

L1 and Hc1 lines, and *clsy1-6*, *nrpd1a-6*, *rdr2-1*, *rdr2-5*, *rdr6-8* mutants have been described before [9], [13], [20], [24].

### Molecular analyses

DNA gel blot analysis, RNA gel blot analysis, and GUS fluorimetric assays were performed as described before. *3′ GUS*, *U6* and *25S* probes have been described before [23], [24], [25].
